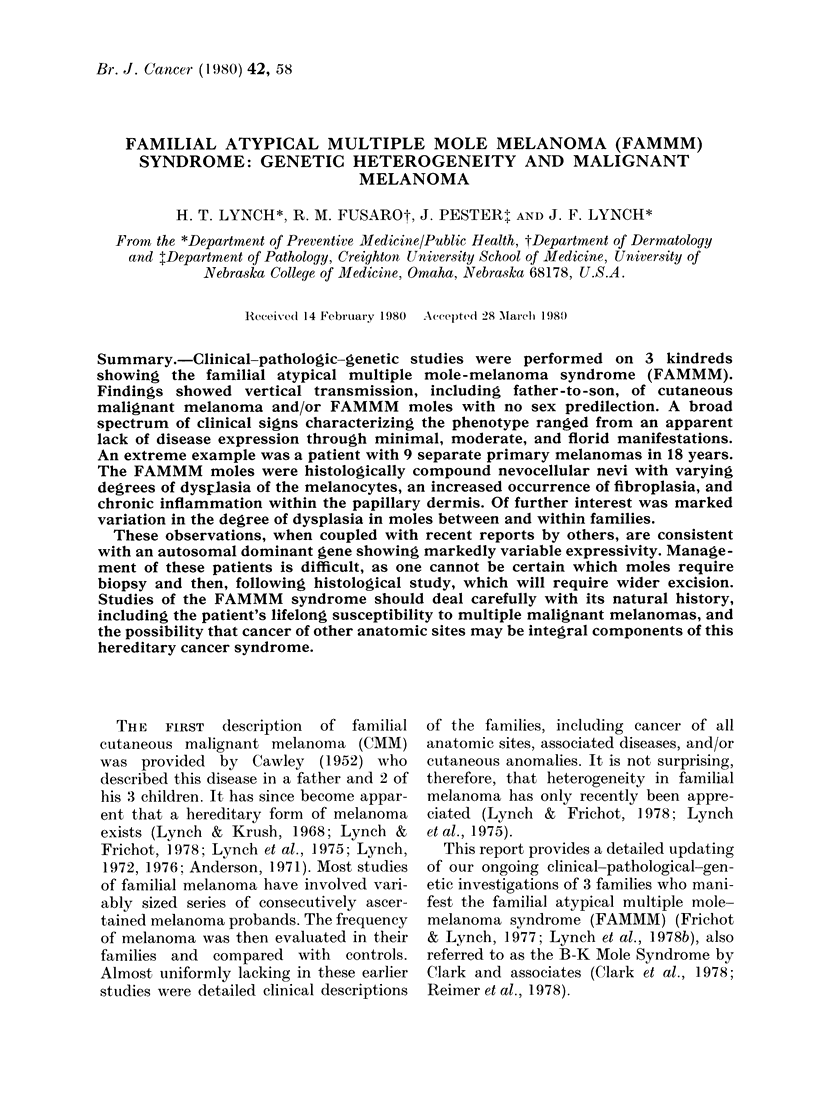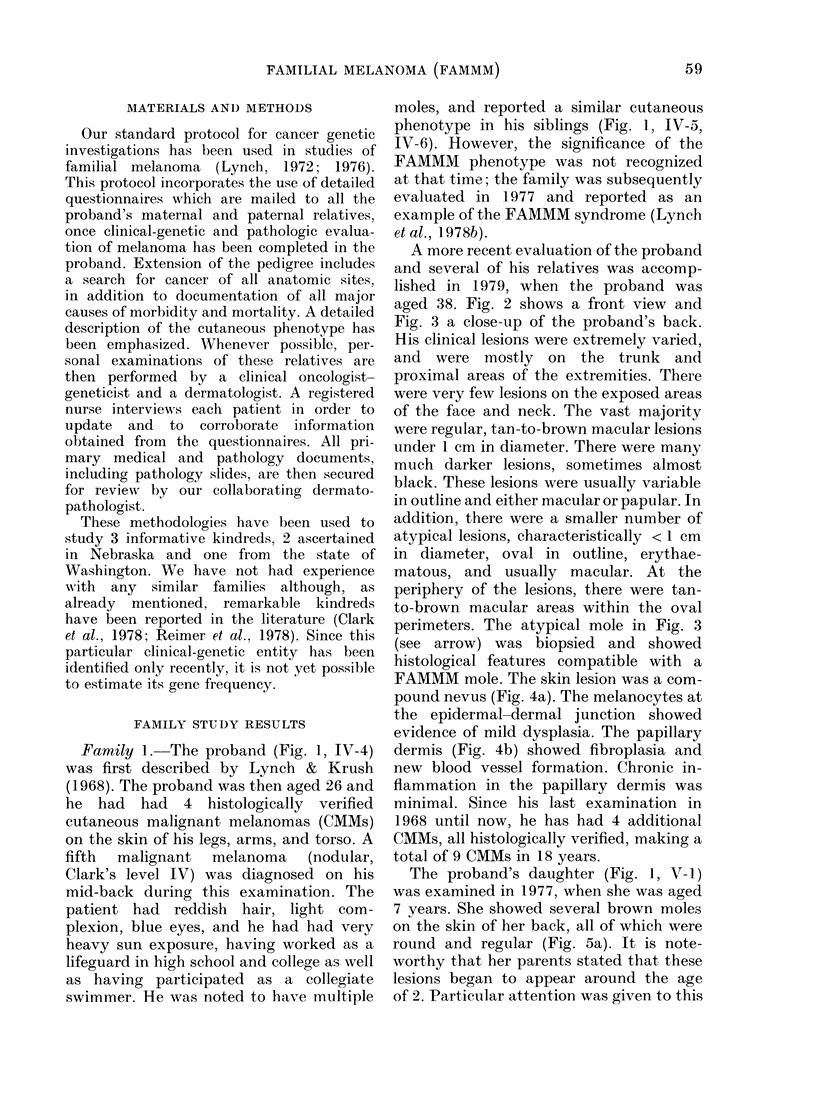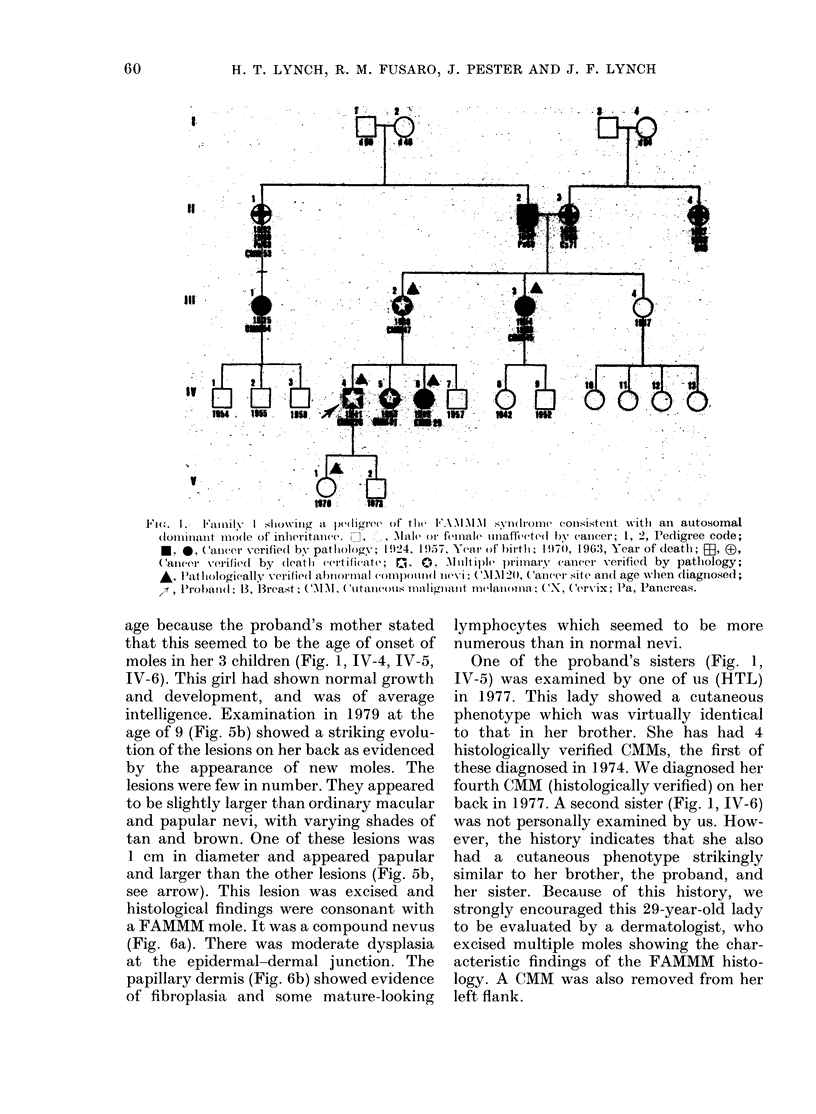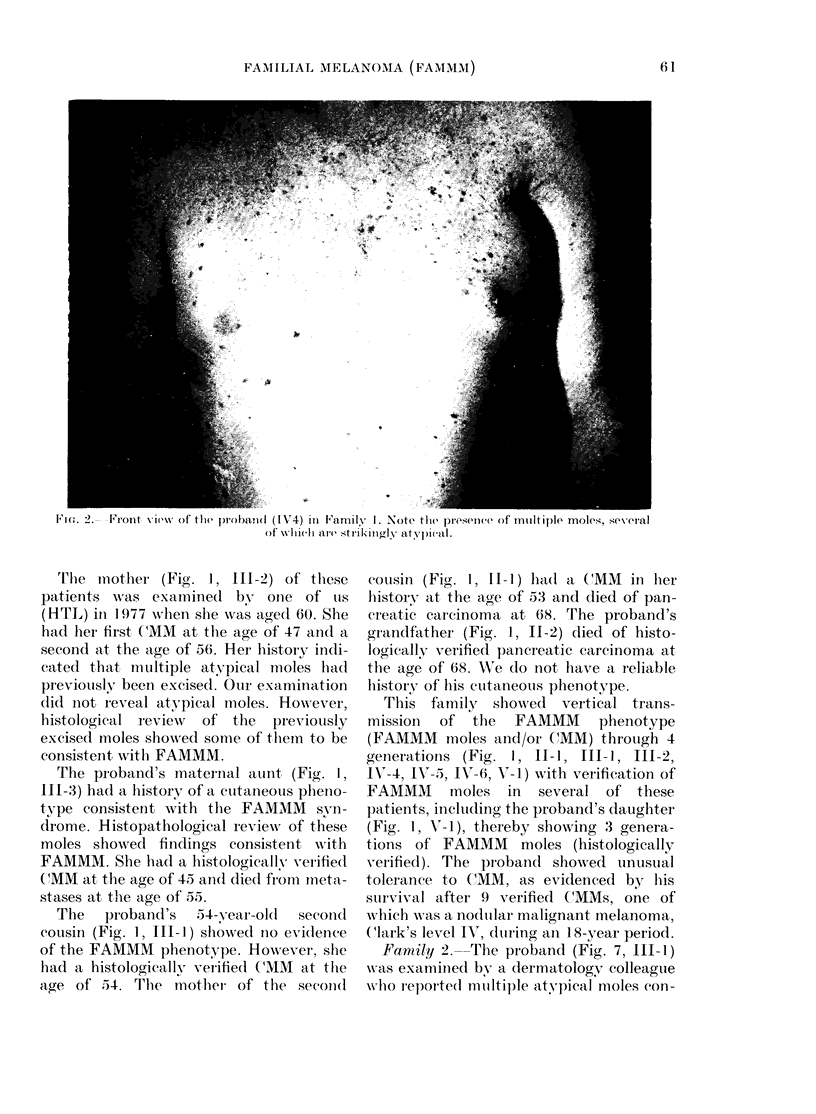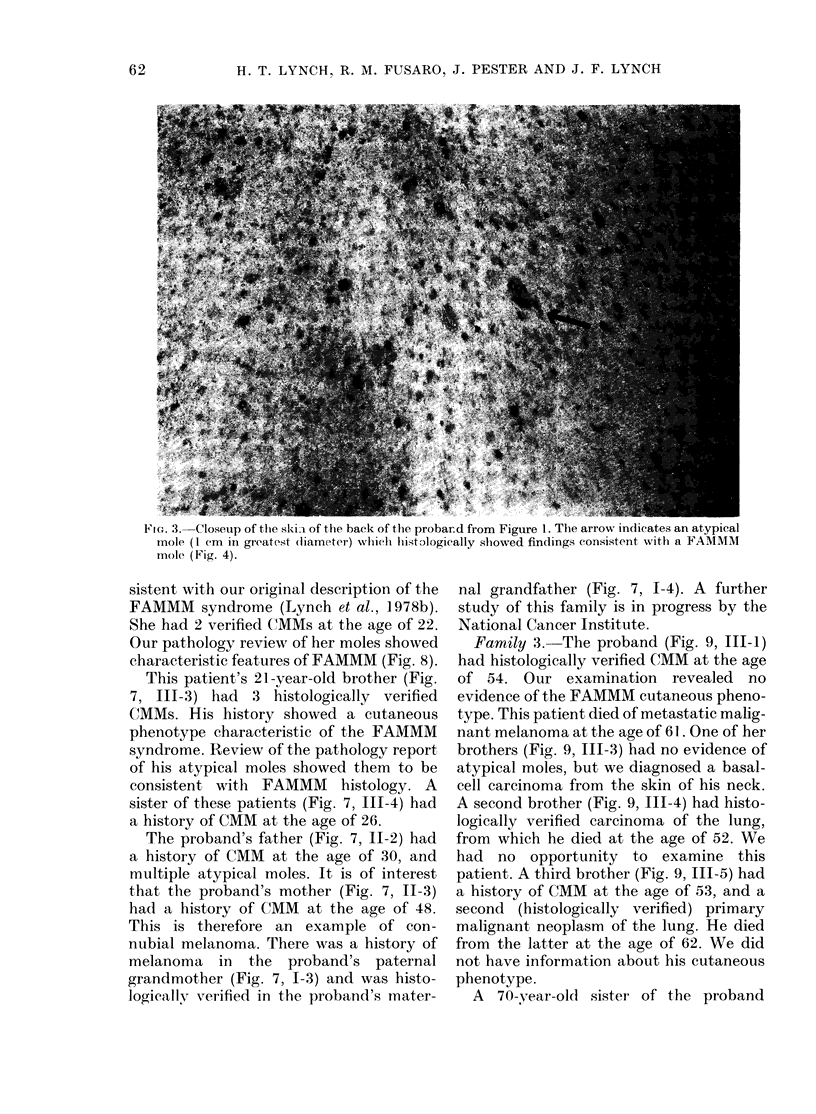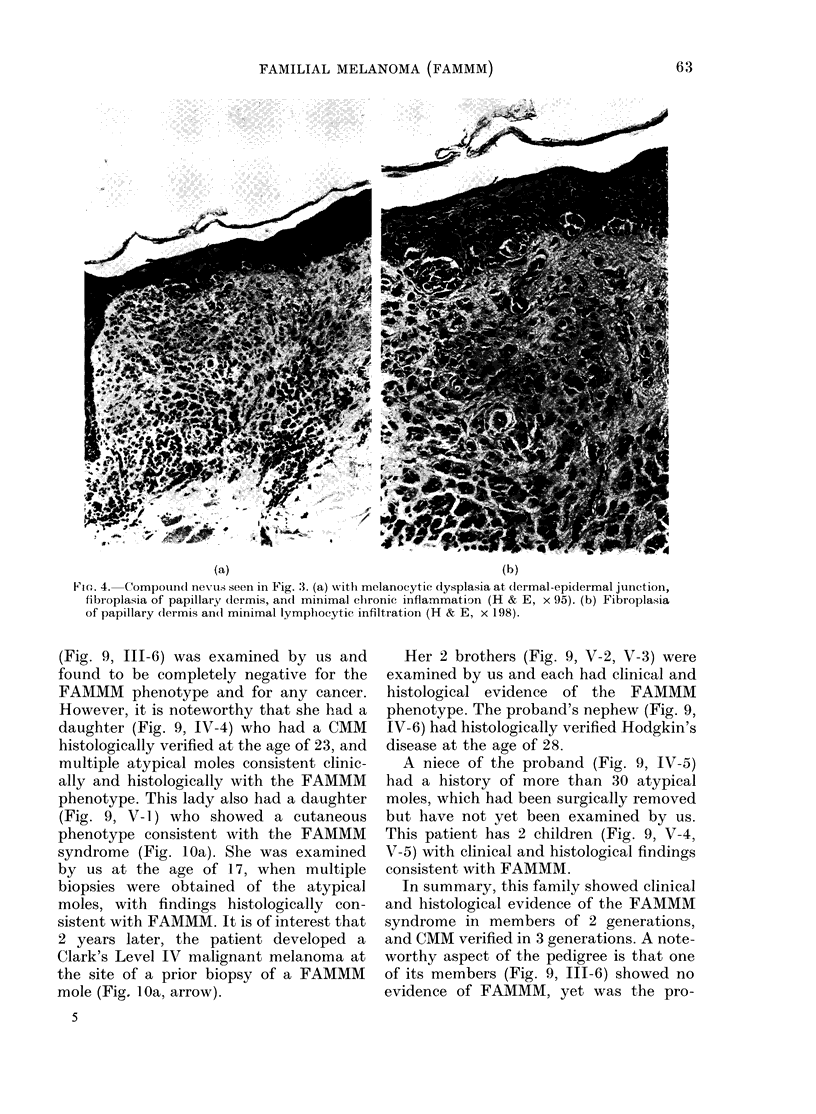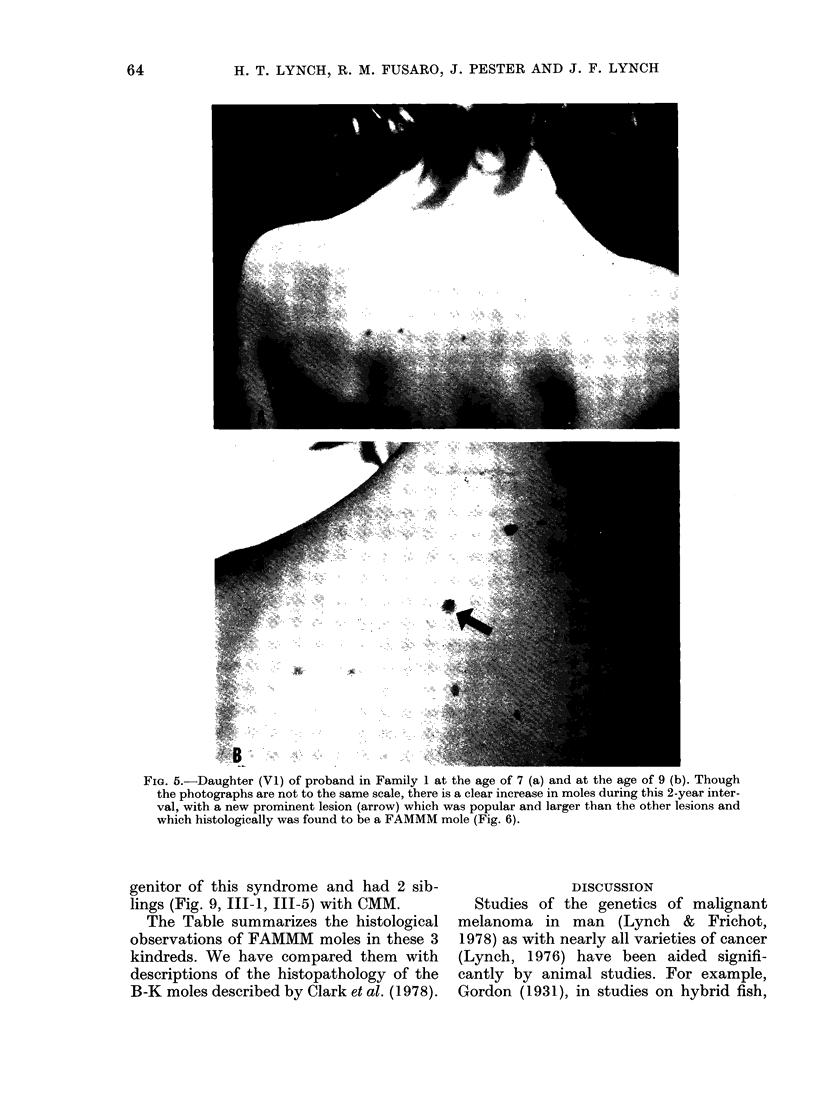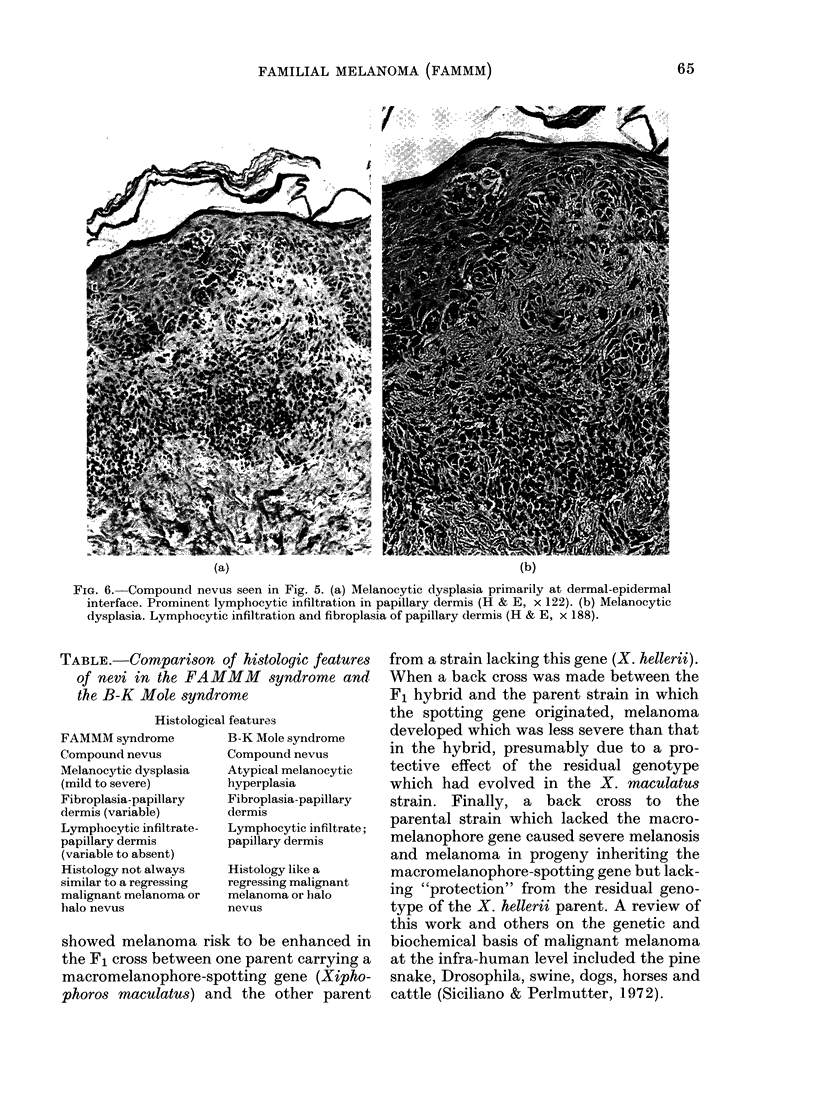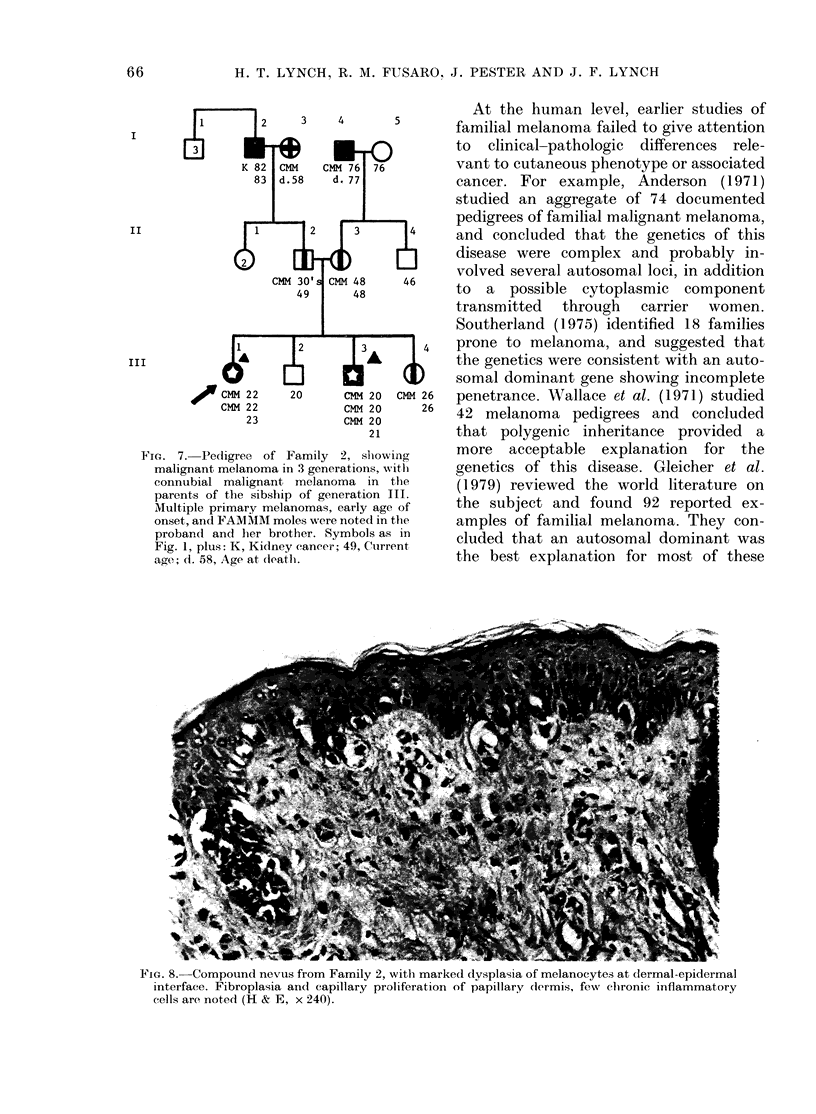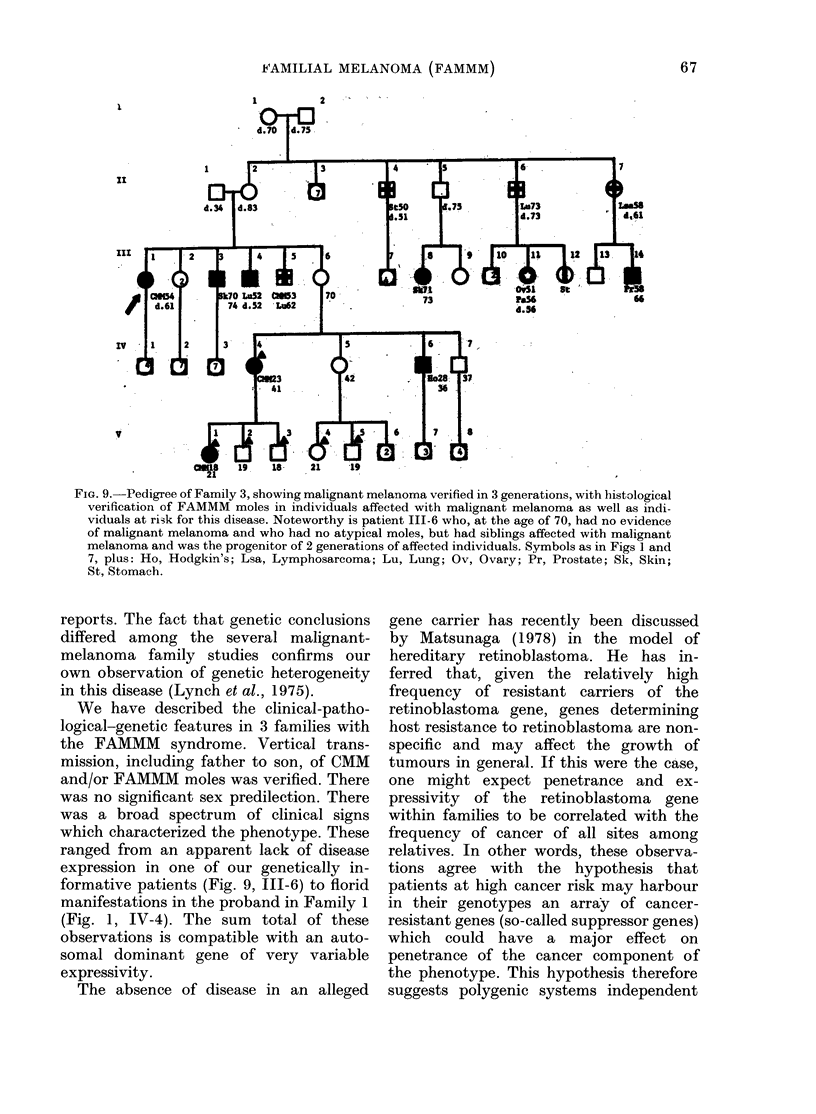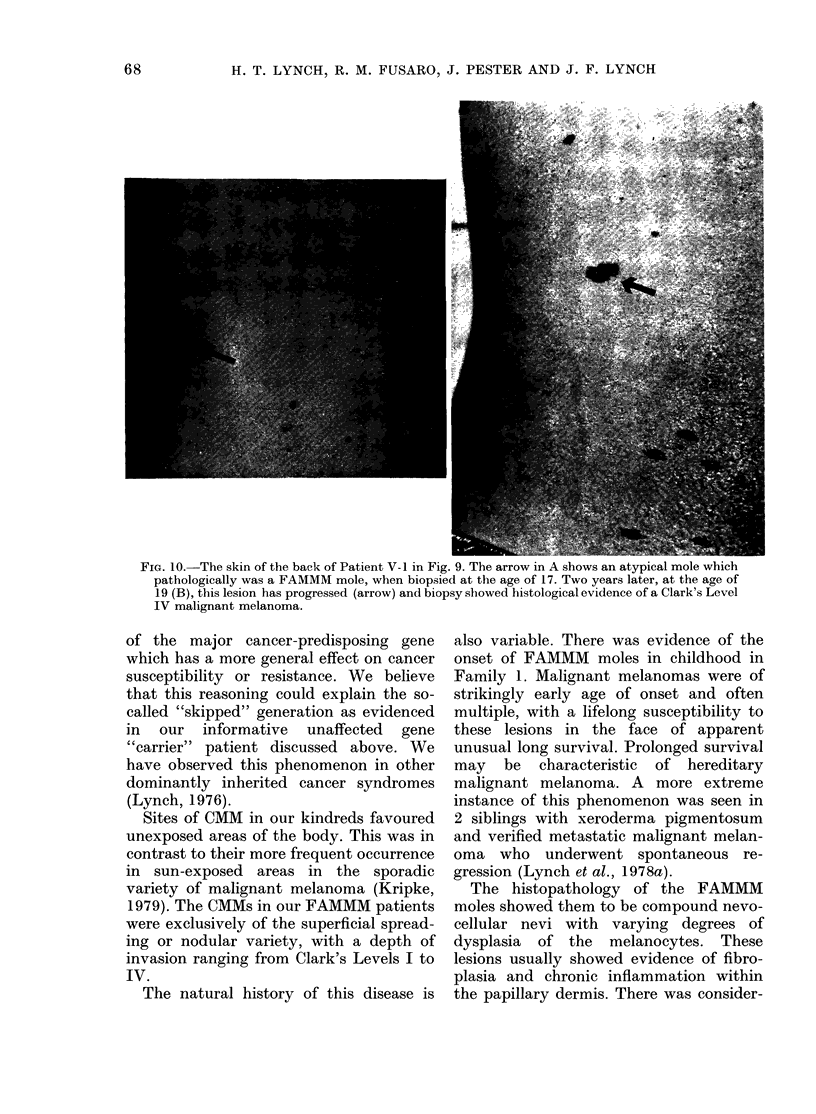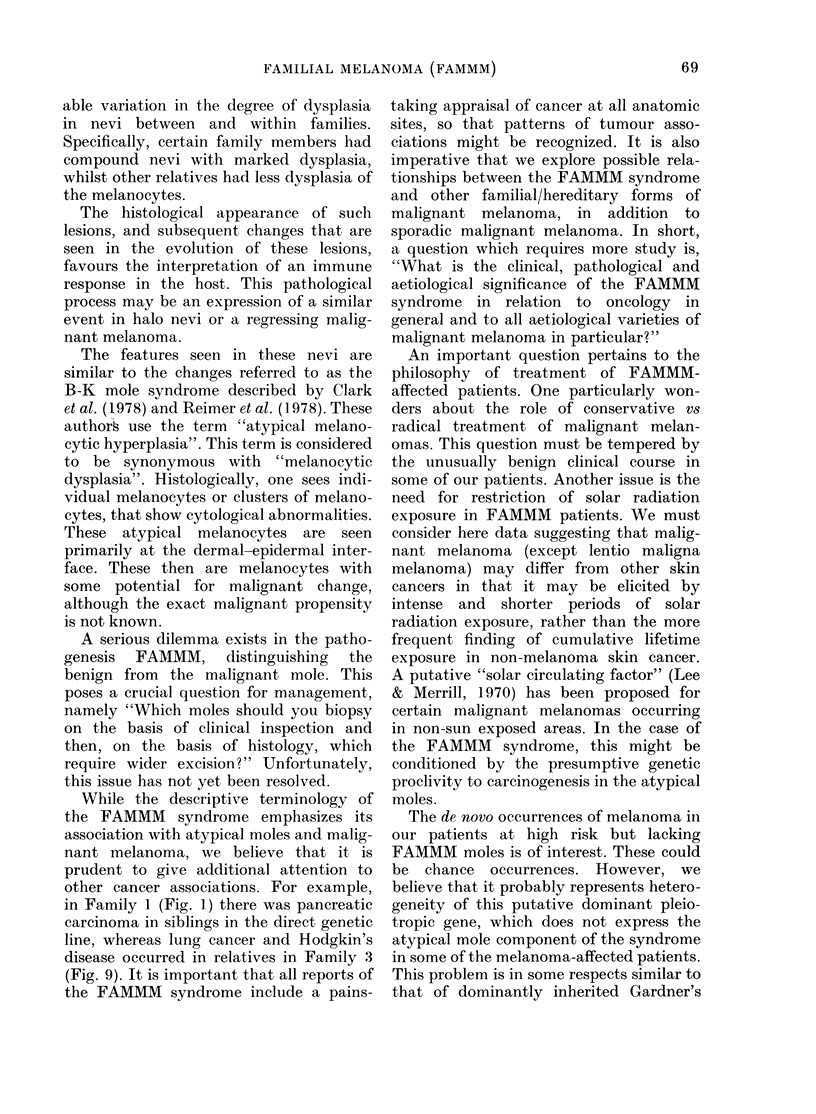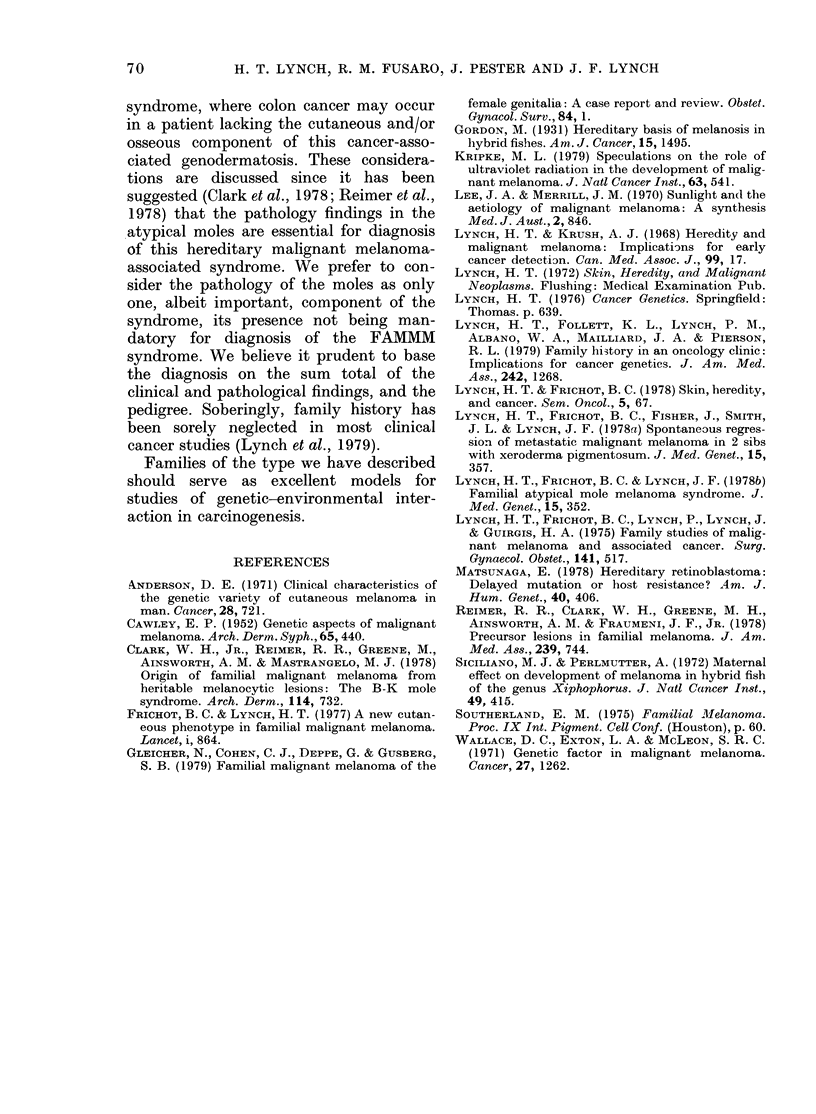# Familial atypical multiple mole melanoma (FAMMM) syndrome: genetic heterogeneity and malignant melanoma.

**DOI:** 10.1038/bjc.1980.203

**Published:** 1980-07

**Authors:** H. T. Lynch, R. M. Fusaro, J. Pester, J. F. Lynch

## Abstract

**Images:**


*Br. J. Cancer* (1980) **42**, 58

### FAMILIAL ATYPICAL MULTIPLE MOLE MELANOMA (FAMMM) SYNDROME: GENETIC HETEROGENEITY AND MALIGNANT MELANOMA

H. T. LYNCH*, R. M. FUSARO†, J. PESTER‡ AND J. F. LYNCH*

*From the *Department of Preventive Medicine/Public Health, †Department of Dermatology and ‡Department of Pathology, Creighton University School of Medicine, University of Nebraska College of Medicine, Omaha, Nebraska* 68178, *U.S.A.*

Received 14 February 1980 Accepted 28 March 1980

**Summary.—Clinical–pathologic–genetic studies were performed on 3 kindreds showing the familial atypical multiple mole-melanoma syndrome (FAMMM). Findings showed vertical transmission, including father-to-son, of cutaneous malignant melanoma and/or FAMMM moles with no sex predilection. A broad spectrum of clinical signs characterizing the phenotype ranged from an apparent lack of disease expression through minimal, moderate, and florid manifestations. An extreme example was a patient with 9 separate primary melanomas in 18 years. The FAMMM moles were histologically compound nevocellular nevi with varying degrees of dysplasia of the melanocytes, an increased occurrence of fibroplasia, and chronic inflammation within the papillary dermis. Of further interest was marked variation in the degree of dysplasia in moles between and within families.**

**These observations, when coupled with recent reports by others, are consistent with an autosomal dominant gene showing markedly variable expressivity. Management of these patients is difficult, as one cannot be certain which moles require biopsy and then, following histological study, which will require wider excision. Studies of the FAMMM syndrome should deal carefully with its natural history, including the patient's lifelong susceptibility to multiple malignant melanomas, and the possibility that cancer of other anatomic sites may be integral components of this hereditary cancer syndrome.**

THE FIRST description of familial cutaneous malignant melanoma (CMM) was provided by Cawley (1952) who described this disease in a father and 2 of his 3 children. It has since become apparent that a hereditary form of melanoma exists (Lynch & Krush, 1968; Lynch & Frichot, 1978; Lynch *et al.*, 1975; Lynch, 1972, 1976; Anderson, 1971). Most studies of familial melanoma have involved variably sized series of consecutively ascertained melanoma probands. The frequency of melanoma was then evaluated in their families and compared with controls. Almost uniformly lacking in these earlier studies were detailed clinical descriptions of the families, including cancer of all anatomic sites, associated diseases, and/or cutaneous anomalies. It is not surprising, therefore, that heterogeneity in familial melanoma has only recently been appreciated (Lynch & Frichot, 1978; Lynch *et al.*, 1975).

This report provides a detailed updating of our ongoing clinical–pathological–genetic investigations of 3 families who manifest the familial atypical multiple mole–melanoma syndrome (FAMMM) (Frichot & Lynch, 1977; Lynch *et al.*, 1978*b*), also referred to as the B-K Mole Syndrome by Clark and associates (Clark *et al.*, 1978; Reimer *et al.*, 1978).

### MATERIALS AND METHODS

Our standard protocol for cancer genetic investigations has been used in studies of familial melanoma (Lynch, 1972; 1976). This protocol incorporates the use of detailed questionnaires which are mailed to all the proband's maternal and paternal relatives, once clinical-genetic and pathologic evaluation of melanoma has been completed in the proband. Extension of the pedigree includes a search for cancer of all anatomic sites, in addition to documentation of all major causes of morbidity and mortality. A detailed description of the cutaneous phenotype has been emphasized. Whenever possible, personal examinations of these relatives are then performed by a clinical oncologist–geneticist and a dermatologist. A registered nurse interviews each patient in order to update and to corroborate information obtained from the questionnaires. All primary medical and pathology documents, including pathology slides, are then secured for review by our collaborating dermatopathologist.

These methodologies have been used to study 3 informative kindreds, 2 ascertained in Nebraska and one from the state of Washington. We have not had experience with any similar families although, as already mentioned, remarkable kindreds have been reported in the literature (Clark *et al.*, 1978; Reimer *et al.*, 1978). Since this particular clinical-genetic entity has been identified only recently, it is not yet possible to estimate its gene frequency.

### FAMILY STUDY RESULTS

*Family* 1.—The proband (Fig. 1, IV-4) was first described by Lynch & Krush (1968). The proband was then aged 26 and he had had 4 histologically verified cutaneous malignant melanomas (CMMs) on the skin of his legs, arms, and torso. A fifth malignant melanoma (nodular, Clark's level IV) was diagnosed on his mid-back during this examination. The patient had reddish hair, light complexion, blue eyes, and he had had very heavy sun exposure, having worked as a lifeguard in high school and college as well as having participated as a collegiate swimmer. He was noted to have multiple moles, and reported a similar cutaneous phenotype in his siblings (Fig. 1, IV-5, IV-6). However, the significance of the FAMMM phenotype was not recognized at that time; the family was subsequently evaluated in 1977 and reported as an example of the FAMMM syndrome (Lynch *et al.*, 1978*b*).

A more recent evaluation of the proband and several of his relatives was accomplished in 1979, when the proband was aged 38. Fig. 2 shows a front view and Fig. 3 a close-up of the proband's back. His clinical lesions were extremely varied, and were mostly on the trunk and proximal areas of the extremities. There were very few lesions on the exposed areas of the face and neck. The vast majority were regular, tan-to-brown macular lesions under 1 cm in diameter. There were many much darker lesions, sometimes almost black. These lesions were usually variable in outline and either macular or papular. In addition, there were a smaller number of atypical lesions, characteristically $< 1$ cm in diameter, oval in outline, erythaematous, and usually macular. At the periphery of the lesions, there were tan-to-brown macular areas within the oval perimeters. The atypical mole in Fig. 3 (see arrow) was biopsied and showed histological features compatible with a FAMMM mole. The skin lesion was a compound nevus (Fig. 4a). The melanocytes at the epidermal–dermal junction showed evidence of mild dysplasia. The papillary dermis (Fig. 4b) showed fibroplasia and new blood vessel formation. Chronic inflammation in the papillary dermis was minimal. Since his last examination in 1968 until now, he has had 4 additional CMMs, all histologically verified, making a total of 9 CMMs in 18 years.

The proband's daughter (Fig. 1, V-1) was examined in 1977, when she was aged 7 years. She showed several brown moles on the skin of her back, all of which were round and regular (Fig. 5a). It is noteworthy that her parents stated that these lesions began to appear around the age of 2. Particular attention was given to this

FIG. 1.- Family I showing a pedigree of the FAMMM syndrome consistent with an autosomal dominant mode of inheritance. □, ○, Male or female unaffected by cancer; 1, 2, Pedigree code; ■, ●, Cancer verified by pathology; 1924, 1957, Year of birth; 1970, 1963, Year of death; ⊞, ⊕, Cancer verified by death certificate; [symbol], ✪, Multiple primary cancer verified by pathology; ▲, Pathologically verified abnormal compound nevi; CMM20, Cancer site and age when diagnosed; ↗, Proband; B, Breast; CMM, Cutaneous malignant melanoma; CX, Cervix; Pa, Pancreas.

age because the proband's mother stated that this seemed to be the age of onset of moles in her 3 children (Fig. 1, IV-4, IV-5, IV-6). This girl had shown normal growth and development, and was of average intelligence. Examination in 1979 at the age of 9 (Fig. 5b) showed a striking evolution of the lesions on her back as evidenced by the appearance of new moles. The lesions were few in number. They appeared to be slightly larger than ordinary macular and papular nevi, with varying shades of tan and brown. One of these lesions was 1 cm in diameter and appeared papular and larger than the other lesions (Fig. 5b, see arrow). This lesion was excised and histological findings were consonant with a FAMMM mole. It was a compound nevus (Fig. 6a). There was moderate dysplasia at the epidermal–dermal junction. The papillary dermis (Fig. 6b) showed evidence of fibroplasia and some mature-looking lymphocytes which seemed to be more numerous than in normal nevi.

One of the proband's sisters (Fig. 1, IV-5) was examined by one of us (HTL) in 1977. This lady showed a cutaneous phenotype which was virtually identical to that in her brother. She has had 4 histologically verified CMMs, the first of these diagnosed in 1974. We diagnosed her fourth CMM (histologically verified) on her back in 1977. A second sister (Fig. 1, IV-6) was not personally examined by us. However, the history indicates that she also had a cutaneous phenotype strikingly similar to her brother, the proband, and her sister. Because of this history, we strongly encouraged this 29-year-old lady to be evaluated by a dermatologist, who excised multiple moles showing the characteristic findings of the FAMMM histology. A CMM was also removed from her left flank.

FIG. 2.—Front view of the proband (IV4) in Family 1. Note the presence of multiple moles, several of which are strikingly atypical.

The mother (Fig. 1, III-2) of these patients was examined by one of us (HTL) in 1977 when she was aged 60. She had her first CMM at the age of 47 and a second at the age of 56. Her history indicated that multiple atypical moles had previously been excised. Our examination did not reveal atypical moles. However, histological review of the previously excised moles showed some of them to be consistent with FAMMM.

The proband's maternal aunt (Fig. 1, III-3) had a history of a cutaneous phenotype consistent with the FAMMM syndrome. Histopathological review of these moles showed findings consistent with FAMMM. She had a histologically verified CMM at the age of 45 and died from metastases at the age of 55.

The proband's 54-year-old second cousin (Fig. 1, III-1) showed no evidence of the FAMMM phenotype. However, she had a histologically verified CMM at the age of 54. The mother of the second cousin (Fig. 1, II-1) had a CMM in her history at the age of 53 and died of pancreatic carcinoma at 68. The proband's grandfather (Fig. 1, II-2) died of histologically verified pancreatic carcinoma at the age of 68. We do not have a reliable history of his cutaneous phenotype.

This family showed vertical transmission of the FAMMM phenotype (FAMMM moles and/or CMM) through 4 generations (Fig. 1, II-1, III-1, III-2, IV-4, IV-5, IV-6, V-1) with verification of FAMMM moles in several of these patients, including the proband's daughter (Fig. 1, V-1), thereby showing 3 generations of FAMMM moles (histologically verified). The proband showed unusual tolerance to CMM, as evidenced by his survival after 9 verified CMMs, one of which was a nodular malignant melanoma, Clark's level IV, during an 18-year period.

*Family* 2.—The proband (Fig. 7, III-1) was examined by a dermatology colleague who reported multiple atypical moles con-

FIG. 3.—Closeup of the skin of the back of the proband from Figure 1. The arrow indicates an atypical mole (1 cm in greatest diameter) which histologically showed findings consistent with a FAMMM mole (Fig. 4).

sistent with our original description of the FAMMM syndrome (Lynch *et al.*, 1978b). She had 2 verified CMMs at the age of 22. Our pathology review of her moles showed characteristic features of FAMMM (Fig. 8).

This patient's 21-year-old brother (Fig. 7, III-3) had 3 histologically verified CMMs. His history showed a cutaneous phenotype characteristic of the FAMMM syndrome. Review of the pathology report of his atypical moles showed them to be consistent with FAMMM histology. A sister of these patients (Fig. 7, III-4) had a history of CMM at the age of 26.

The proband's father (Fig. 7, II-2) had a history of CMM at the age of 30, and multiple atypical moles. It is of interest that the proband's mother (Fig. 7, II-3) had a history of CMM at the age of 48. This is therefore an example of connubial melanoma. There was a history of melanoma in the proband's paternal grandmother (Fig. 7, I-3) and was histologically verified in the proband's maternal grandfather (Fig. 7, I-4). A further study of this family is in progress by the National Cancer Institute.

*Family* 3.—The proband (Fig. 9, III-1) had histologically verified CMM at the age of 54. Our examination revealed no evidence of the FAMMM cutaneous phenotype. This patient died of metastatic malignant melanoma at the age of 61. One of her brothers (Fig. 9, III-3) had no evidence of atypical moles, but we diagnosed a basal-cell carcinoma from the skin of his neck. A second brother (Fig. 9, III-4) had histologically verified carcinoma of the lung, from which he died at the age of 52. We had no opportunity to examine this patient. A third brother (Fig. 9, III-5) had a history of CMM at the age of 53, and a second (histologically verified) primary malignant neoplasm of the lung. He died from the latter at the age of 62. We did not have information about his cutaneous phenotype.

A 70-year-old sister of the proband

FIG. 4.—Compound nevus seen in Fig. 3. (a) with melanocytic dysplasia at dermal-epidermal junction, fibroplasia of papillary dermis, and minimal chronic inflammation (H & E, × 95). (b) Fibroplasia of papillary dermis and minimal lymphocytic infiltration (H & E, × 198).

(Fig. 9, III-6) was examined by us and found to be completely negative for the FAMMM phenotype and for any cancer. However, it is noteworthy that she had a daughter (Fig. 9, IV-4) who had a CMM histologically verified at the age of 23, and multiple atypical moles consistent clinically and histologically with the FAMMM phenotype. This lady also had a daughter (Fig. 9, V-1) who showed a cutaneous phenotype consistent with the FAMMM syndrome (Fig. 10a). She was examined by us at the age of 17, when multiple biopsies were obtained of the atypical moles, with findings histologically consistent with FAMMM. It is of interest that 2 years later, the patient developed a Clark's Level IV malignant melanoma at the site of a prior biopsy of a FAMMM mole (Fig. 10a, arrow).

Her 2 brothers (Fig. 9, V-2, V-3) were examined by us and each had clinical and histological evidence of the FAMMM phenotype. The proband's nephew (Fig. 9, IV-6) had histologically verified Hodgkin's disease at the age of 28.

A niece of the proband (Fig. 9, IV-5) had a history of more than 30 atypical moles, which had been surgically removed but have not yet been examined by us. This patient has 2 children (Fig. 9, V-4, V-5) with clinical and histological findings consistent with FAMMM.

In summary, this family showed clinical and histological evidence of the FAMMM syndrome in members of 2 generations, and CMM verified in 3 generations. A noteworthy aspect of the pedigree is that one of its members (Fig. 9, III-6) showed no evidence of FAMMM, yet was the pro-

FIG. 5.—Daughter (VI) of proband in Family 1 at the age of 7 (a) and at the age of 9 (b). Though the photographs are not to the same scale, there is a clear increase in moles during this 2-year interval, with a new prominent lesion (arrow) which was popular and larger than the other lesions and which histologically was found to be a FAMMM mole (Fig. 6).

genitor of this syndrome and had 2 siblings (Fig. 9, III-1, III-5) with CMM.

The Table summarizes the histological observations of FAMMM moles in these 3 kindreds. We have compared them with descriptions of the histopathology of the B-K moles described by Clark *et al.* (1978).

### DISCUSSION

Studies of the genetics of malignant melanoma in man (Lynch & Frichot, 1978) as with nearly all varieties of cancer (Lynch, 1976) have been aided significantly by animal studies. For example, Gordon (1931), in studies on hybrid fish,

FIG. 6.—Compound nevus seen in Fig. 5. (a) Melanocytic dysplasia primarily at dermal-epidermal interface. Prominent lymphocytic infiltration in papillary dermis (H & E, × 122). (b) Melanocytic dysplasia. Lymphocytic infiltration and fibroplasia of papillary dermis (H & E, × 188).

TABLE.—*Comparison of histologic features of nevi in the FAMMM syndrome and the B-K Mole syndrome*

| Histological features | |
|---|---|
| FAMMM syndrome | B-K Mole syndrome |
| Compound nevus | Compound nevus |
| Melanocytic dysplasia (mild to severe) | Atypical melanocytic hyperplasia |
| Fibroplasia-papillary dermis (variable) | Fibroplasia-papillary dermis |
| Lymphocytic infiltrate-papillary dermis (variable to absent) | Lymphocytic infiltrate; papillary dermis |
| Histology not always similar to a regressing malignant melanoma or halo nevus | Histology like a regressing malignant melanoma or halo nevus |

showed melanoma risk to be enhanced in the $F_1$ cross between one parent carrying a macromelanophore-spotting gene (*Xiphophoros maculatus*) and the other parent from a strain lacking this gene (*X. hellerii*). When a back cross was made between the $F_1$ hybrid and the parent strain in which the spotting gene originated, melanoma developed which was less severe than that in the hybrid, presumably due to a protective effect of the residual genotype which had evolved in the *X. maculatus* strain. Finally, a back cross to the parental strain which lacked the macromelanophore gene caused severe melanosis and melanoma in progeny inheriting the macromelanophore-spotting gene but lacking "protection" from the residual genotype of the *X. hellerii* parent. A review of this work and others on the genetic and biochemical basis of malignant melanoma at the infra-human level included the pine snake, Drosophila, swine, dogs, horses and cattle (Siciliano & Perlmutter, 1972).

FIG. 7.—Pedigree of Family 2, showing malignant melanoma in 3 generations, with connubial malignant melanoma in the parents of the sibship of generation III. Multiple primary melanomas, early age of onset, and FAMMM moles were noted in the proband and her brother. Symbols as in Fig. 1, plus: K, Kidney cancer; 49, Current age; d. 58, Age at death.

At the human level, earlier studies of familial melanoma failed to give attention to clinical–pathologic differences relevant to cutaneous phenotype or associated cancer. For example, Anderson (1971) studied an aggregate of 74 documented pedigrees of familial malignant melanoma, and concluded that the genetics of this disease were complex and probably involved several autosomal loci, in addition to a possible cytoplasmic component transmitted through carrier women. Southerland (1975) identified 18 families prone to melanoma, and suggested that the genetics were consistent with an autosomal dominant gene showing incomplete penetrance. Wallace *et al.* (1971) studied 42 melanoma pedigrees and concluded that polygenic inheritance provided a more acceptable explanation for the genetics of this disease. Gleicher *et al.* (1979) reviewed the world literature on the subject and found 92 reported examples of familial melanoma. They concluded that an autosomal dominant was the best explanation for most of these

FIG. 8.—Compound nevus from Family 2, with marked dysplasia of melanocytes at dermal-epidermal interface. Fibroplasia and capillary proliferation of papillary dermis, few chronic inflammatory cells are noted (H & E, × 240).

FIG. 9.—Pedigree of Family 3, showing malignant melanoma verified in 3 generations, with histological verification of FAMMM moles in individuals affected with malignant melanoma as well as individuals at risk for this disease. Noteworthy is patient III-6 who, at the age of 70, had no evidence of malignant melanoma and who had no atypical moles, but had siblings affected with malignant melanoma and was the progenitor of 2 generations of affected individuals. Symbols as in Figs 1 and 7, plus: Ho, Hodgkin's; Lsa, Lymphosarcoma; Lu, Lung; Ov, Ovary; Pr, Prostate; Sk, Skin; St, Stomach.

reports. The fact that genetic conclusions differed among the several malignant-melanoma family studies confirms our own observation of genetic heterogeneity in this disease (Lynch *et al.*, 1975).

We have described the clinical-pathological–genetic features in 3 families with the FAMMM syndrome. Vertical transmission, including father to son, of CMM and/or FAMMM moles was verified. There was no significant sex predilection. There was a broad spectrum of clinical signs which characterized the phenotype. These ranged from an apparent lack of disease expression in one of our genetically informative patients (Fig. 9, III-6) to florid manifestations in the proband in Family 1 (Fig. 1, IV-4). The sum total of these observations is compatible with an autosomal dominant gene of very variable expressivity.

The absence of disease in an alleged gene carrier has recently been discussed by Matsunaga (1978) in the model of hereditary retinoblastoma. He has inferred that, given the relatively high frequency of resistant carriers of the retinoblastoma gene, genes determining host resistance to retinoblastoma are non-specific and may affect the growth of tumours in general. If this were the case, one might expect penetrance and expressivity of the retinoblastoma gene within families to be correlated with the frequency of cancer of all sites among relatives. In other words, these observations agree with the hypothesis that patients at high cancer risk may harbour in their genotypes an array of cancer-resistant genes (so-called suppressor genes) which could have a major effect on penetrance of the cancer component of the phenotype. This hypothesis therefore suggests polygenic systems independent

FIG. 10.—The skin of the back of Patient V-1 in Fig. 9. The arrow in A shows an atypical mole which pathologically was a FAMMM mole, when biopsied at the age of 17. Two years later, at the age of 19 (B), this lesion has progressed (arrow) and biopsy showed histological evidence of a Clark's Level IV malignant melanoma.

of the major cancer-predisposing gene which has a more general effect on cancer susceptibility or resistance. We believe that this reasoning could explain the so-called "skipped" generation as evidenced in our informative unaffected gene "carrier" patient discussed above. We have observed this phenomenon in other dominantly inherited cancer syndromes (Lynch, 1976).

Sites of CMM in our kindreds favoured unexposed areas of the body. This was in contrast to their more frequent occurrence in sun-exposed areas in the sporadic variety of malignant melanoma (Kripke, 1979). The CMMs in our FAMMM patients were exclusively of the superficial spreading or nodular variety, with a depth of invasion ranging from Clark's Levels I to IV.

The natural history of this disease is also variable. There was evidence of the onset of FAMMM moles in childhood in Family 1. Malignant melanomas were of strikingly early age of onset and often multiple, with a lifelong susceptibility to these lesions in the face of apparent unusual long survival. Prolonged survival may be characteristic of hereditary malignant melanoma. A more extreme instance of this phenomenon was seen in 2 siblings with xeroderma pigmentosum and verified metastatic malignant melanoma who underwent spontaneous regression (Lynch *et al.*, 1978*a*).

The histopathology of the FAMMM moles showed them to be compound nevocellular nevi with varying degrees of dysplasia of the melanocytes. These lesions usually showed evidence of fibroplasia and chronic inflammation within the papillary dermis. There was considerable variation in the degree of dysplasia in nevi between and within families. Specifically, certain family members had compound nevi with marked dysplasia, whilst other relatives had less dysplasia of the melanocytes.

The histological appearance of such lesions, and subsequent changes that are seen in the evolution of these lesions, favours the interpretation of an immune response in the host. This pathological process may be an expression of a similar event in halo nevi or a regressing malignant melanoma.

The features seen in these nevi are similar to the changes referred to as the B-K mole syndrome described by Clark *et al.* (1978) and Reimer *et al.* (1978). These authors use the term "atypical melanocytic hyperplasia". This term is considered to be synonymous with "melanocytic dysplasia". Histologically, one sees individual melanocytes or clusters of melanocytes, that show cytological abnormalities. These atypical melanocytes are seen primarily at the dermal–epidermal interface. These then are melanocytes with some potential for malignant change, although the exact malignant propensity is not known.

A serious dilemma exists in the pathogenesis FAMMM, distinguishing the benign from the malignant mole. This poses a crucial question for management, namely "Which moles should you biopsy on the basis of clinical inspection and then, on the basis of histology, which require wider excision?" Unfortunately, this issue has not yet been resolved.

While the descriptive terminology of the FAMMM syndrome emphasizes its association with atypical moles and malignant melanoma, we believe that it is prudent to give additional attention to other cancer associations. For example, in Family 1 (Fig. 1) there was pancreatic carcinoma in siblings in the direct genetic line, whereas lung cancer and Hodgkin's disease occurred in relatives in Family 3 (Fig. 9). It is important that all reports of the FAMMM syndrome include a painstaking appraisal of cancer at all anatomic sites, so that patterns of tumour associations might be recognized. It is also imperative that we explore possible relationships between the FAMMM syndrome and other familial/hereditary forms of malignant melanoma, in addition to sporadic malignant melanoma. In short, a question which requires more study is, "What is the clinical, pathological and aetiological significance of the FAMMM syndrome in relation to oncology in general and to all aetiological varieties of malignant melanoma in particular?"

An important question pertains to the philosophy of treatment of FAMMM-affected patients. One particularly wonders about the role of conservative *vs* radical treatment of malignant melanomas. This question must be tempered by the unusually benign clinical course in some of our patients. Another issue is the need for restriction of solar radiation exposure in FAMMM patients. We must consider here data suggesting that malignant melanoma (except lentio maligna melanoma) may differ from other skin cancers in that it may be elicited by intense and shorter periods of solar radiation exposure, rather than the more frequent finding of cumulative lifetime exposure in non-melanoma skin cancer. A putative "solar circulating factor" (Lee & Merrill, 1970) has been proposed for certain malignant melanomas occurring in non-sun exposed areas. In the case of the FAMMM syndrome, this might be conditioned by the presumptive genetic proclivity to carcinogenesis in the atypical moles.

The *de novo* occurrences of melanoma in our patients at high risk but lacking FAMMM moles is of interest. These could be chance occurrences. However, we believe that it probably represents heterogeneity of this putative dominant pleiotropic gene, which does not express the atypical mole component of the syndrome in some of the melanoma-affected patients. This problem is in some respects similar to that of dominantly inherited Gardner's syndrome, where colon cancer may occur in a patient lacking the cutaneous and/or osseous component of this cancer-associated genodermatosis. These considerations are discussed since it has been suggested (Clark *et al.*, 1978; Reimer *et al.*, 1978) that the pathology findings in the atypical moles are essential for diagnosis of this hereditary malignant melanoma-associated syndrome. We prefer to consider the pathology of the moles as only one, albeit important, component of the syndrome, its presence not being mandatory for diagnosis of the FAMMM syndrome. We believe it prudent to base the diagnosis on the sum total of the clinical and pathological findings, and the pedigree. Soberingly, family history has been sorely neglected in most clinical cancer studies (Lynch *et al.*, 1979).

Families of the type we have described should serve as excellent models for studies of genetic–environmental interaction in carcinogenesis.

### REFERENCES

ANDERSON, D. E. (1971) Clinical characteristics of the genetic variety of cutaneous melanoma in man. *Cancer*, **28**, 721.

CAWLEY, E. P. (1952) Genetic aspects of malignant melanoma. *Arch. Derm. Syph.*, **65**, 440.

CLARK, W. H., JR., REIMER, R. R., GREENE, M., AINSWORTH, A. M. & MASTRANGELO, M. J. (1978) Origin of familial malignant melanoma from heritable melanocytic lesions: The B-K mole syndrome. *Arch. Derm.*, **114**, 732.

FRICHOT, B. C. & LYNCH, H. T. (1977) A new cutaneous phenotype in familial malignant melanoma. *Lancet*, i, 864.

GLEICHER, N., COHEN, C. J., DEPPE, G. & GUSBERG, S. B. (1979) Familial malignant melanoma of the female genitalia: A case report and review. *Obstet. Gynacol. Surv.*, **84**, 1.

GORDON, M. (1931) Hereditary basis of melanosis in hybrid fishes. *Am. J. Cancer*, **15**, 1495.

KRIPKE, M. L. (1979) Speculations on the role of ultraviolet radiation in the development of malignant melanoma. *J. Natl Cancer Inst.*, **63**, 541.

LEE, J. A. & MERRILL, J. M. (1970) Sunlight and the aetiology of malignant melanoma: A synthesis *Med. J. Aust.*, **2**, 846.

LYNCH, H. T. & KRUSH, A. J. (1968) Heredity and malignant melanoma: Implications for early cancer detection. *Can. Med. Assoc. J.*, **99**, 17.

LYNCH, H. T. (1972) *Skin, Heredity, and Malignant Neoplasms.* Flushing: Medical Examination Pub.

LYNCH, H. T. (1976) *Cancer Genetics.* Springfield: Thomas. p. 639.

LYNCH, H. T., FOLLETT, K. L., LYNCH, P. M., ALBANO, W. A., MAILLIARD, J. A. & PIERSON, R. L. (1979) Family history in an oncology clinic: Implications for cancer genetics. *J. Am. Med. Ass.*, **242**, 1268.

LYNCH, H. T. & FRICHOT, B. C. (1978) Skin, heredity, and cancer. *Sem. Oncol.*, **5**, 67.

LYNCH, H. T., FRICHOT, B. C., FISHER, J., SMITH, J. L. & LYNCH, J. F. (1978*a*) Spontaneous regression of metastatic malignant melanoma in 2 sibs with xeroderma pigmentosum. *J. Med. Genet.*, **15**, 357.

LYNCH, H. T., FRICHOT, B. C. & LYNCH, J. F. (1978*b*) Familial atypical mole melanoma syndrome. *J. Med. Genet.*, **15**, 352.

LYNCH, H. T., FRICHOT, B. C., LYNCH, P., LYNCH, J. & GUIRGIS, H. A. (1975) Family studies of malignant melanoma and associated cancer. *Surg. Gynaecol. Obstet.*, **141**, 517.

MATSUNAGA, E. (1978) Hereditary retinoblastoma: Delayed mutation or host resistance? *Am. J. Hum. Genet.*, **40**, 406.

REIMER, R. R., CLARK, W. H., GREENE, M. H., AINSWORTH, A. M. & FRAUMENI, J. F., JR. (1978) Precursor lesions in familial melanoma. *J. Am. Med. Ass.*, **239**, 744.

SICILIANO, M. J. & PERLMUTTER, A. (1972) Maternal effect on development of melanoma in hybrid fish of the genus *Xiphophorus*. *J. Natl Cancer Inst.*, **49**, 415.

SOUTHERLAND, E. M. (1975) *Familial Melanoma. Proc. IX Int. Pigment. Cell Conf.* (Houston), p. 60.

WALLACE, D. C., EXTON, L. A. & MCLEON, S. R. C. (1971) Genetic factor in malignant melanoma. *Cancer*, **27**, 1262.